# Development of a Method for Simultaneous Analysis of Allergenic Flavoring Agents in Cigarettes and Quantitative Risk Assessment for Consumer Safety

**DOI:** 10.3390/toxics9040087

**Published:** 2021-04-18

**Authors:** Dae Yong Jang, Hyung Soo Kim, Eun Chul Pack, Ye Ji Koo, Kyung Min Lim, Dal Woong Choi

**Affiliations:** 1 Department of Public Health Science, Graduate School, Korea University, Seoul 02841, Korea; foxrice2@naver.com (D.Y.J.); dkss9900@naver.com (H.S.K.); qordmscjf@nate.com (E.C.P.); 5270463@hanmail.net (Y.J.K.); 2 Health Science Research Center, Korea University, Seoul 02841, Korea; 3 School of Health and Environmental Sciences, Korea University, Seoul 02841, Korea; 4 College of Pharmacy, Ewha Womans University, Seoul 03670, Korea; 5 Department of Health and Safety Convergence Science, Transdisciplinary Major in Learning Health Systems, Graduate School, Korea University, Seoul 02841, Korea

**Keywords:** capsule cigarette, allergenic flavoring agent, gas chromatography-tandem mass spectrometry, inhalation exposure, risk assessment

## Abstract

Flavoring agents are added to cigarettes to improve taste. There are mostly permitted food additives, but some of them are restricted for use in food, cosmetics, and toys, since they can cause allergic reactions. Previous studies have investigated the levels of flavoring agents in tobacco but none has focused on their content in filter tips and capsules. Moreover, no studies have assessed the risk of adding allergenic flavoring agents in cigarettes. Here, we developed and validated a simultaneous analysis method for 25 allergenic flavoring agents and menthol with gas chromatography–tandem mass spectrometry to determine levels of flavoring agents in the tobacco, filter tips, and capsules of 54 commercial cigarettes in Korea. All cigarettes contained at least one allergenic flavoring agent regardless of the inclusion of flavoring capsules. Importantly, the filter tips and the capsules contained higher levels of flavoring agents than tobacco, highlighting the importance of the quantification of flavoring agents in these parts of cigarettes. Nevertheless, the risk assessment based on their levels in cigarettes suggested that their exposure was maintained at a safe level. However, the risk assessed from maximum menthol, linalool, and cinnamaldehyde exceeded one-tenth of derived no-effect levels, suggesting the need for further studies on their risk to human health.

## 1. Introduction

Recently, flavored tobacco products such as cigarettes, cigarillos, cigars, hookah tobacco, and several types of smokeless tobacco, have been gaining popularity especially among youths. Various flavoring agents and additives are added to cigarettes to improve their flavor and thus attract smokers. Most of them have been evaluated for safety by the Flavor and Extract Manufacturers Association and the Joint FAO/WHO Expert Committee on Food Additives (JECFA), and some of them have also been included in the “Substances Added to Food inventory” of the Food and Drug Administration (FDA) [1,2]. However, cigarettes and food are consumed differently, their exposure routes differing from one another, and substances contained in cigarettes may be thermally decomposed or degraded through combustion, unlike in food. Therefore, it is unreasonable to apply the safety criteria of food to cigarettes [3,4]. Additionally, among the substances on the list of JECFA and FDA food additives, certain substances are prohibited from being added to food, cosmetics, and toys in Europe because they pose a risk of causing allergic reactions [5,6,7]. Methyl eugenol was removed from the list of “Generally Recognized as Safe” items since the 28th update of the Flavor and Extract Manufacturers Association [8], and Regulation (EC) No 1334/2008 classifies methyl eugenol and coumarin as “Substances which shall not be added as such to food” [9]. In addition, the FDA removed methyl eugenol from the food additive list owing to its carcinogenic effects in experimental animals [10] and classified coumarin as “Generally Prohibited from Direct Addition or Use as Human Food” [11]. Lilial and hydroxyisohexyl 3-cyclohexene carboxaldehyde (HICC) are not included in food additives of the FDA and JECFA, and HICC should not be used in cosmetics, as it is a fragrance allergen that has frequently caused contact allergies [12].

As described above, the use of flavoring agents known to cause allergic reactions is regulated in various fields, but tobacco products are not regulated for these flavoring agents. In fact, flavoring agents were generally considered relatively safer than the other harmful substances contained in cigarettes. Thus, they have received less attention, and accordingly, there is relatively little research or data on the analysis of flavoring agents. To summarize previous findings on flavoring agents in cigarettes, most studies were limited to the tobacco component of tobacco-related products, and the research methodologies were limited to screening for substances contained in tobacco [13], comparison of the extraction efficiency of various extraction methods (e.g., solvent extraction, Soxhlet, and ultrasonication [14]), and development of a quantitative method and its validation for specific additives including flavoring agents [15,16,17]. Even in these studies, few have been conducted on allergenic flavorings. Moreover, the analytical studies on cigarettes are mainly focused on the ingredients added to tobacco. In addition, quantitative risk assessment has not been done based on the content of flavoring agents in tobacco products. Here, we focused on the analysis of allergenic flavoring agents contained in the components of cigarettes other than tobacco, and their risk to human health.

In this study, we developed and validated a gas chromatography–tandem mass spectrometry (GC-MS/MS) method to simultaneously analyze 25 allergenic flavoring agents (restricted for use in food, cosmetics, and toys in Europe) and menthol (the most common flavoring agent added to cigarettes) in tobacco, filter tips, and capsules in cigarettes. In addition, we used this analysis method to quantify the levels of flavoring agents in commercial cigarettes and assessed the exposure levels through inhalation associated with the absorption of these flavorings resulting from smoking the flavored cigarettes. Additionally, exposure levels through inhalation were compared with the derived no-effect level (DNEL) of the International Uniform Chemical Information Database version 6 to quantitatively assess the risk of allergenic flavoring agents to smokers of flavored cigarettes [18].

## 2. Materials and Methods

### 2.1. Chemicals

The standards α-amylcinnamaldehyde (CAS RN 122-40-7, purity ≥97.0%), α-amylcinnamyl alcohol (CAS RN 101-85-9, purity ≥98.0%), cinnamaldehyde (CAS RN 104-55-2, purity ≥95%), cinnamyl alcohol (CAS RN 104-54-1, purity ≥98.0%), α−isomethyl ionone (CAS RN 127-51-5, purity ≥95%), HICC (CAS RN 31906-04-4, purity ≥97.0%), hexyl cinnamaldehyde (CAS RN 101-86-0, purity ≥95%), hydroxycitronellal (CAS RN 107-75-5, purity ≥95%), farnesol (CAS RN 4602-84-0, purity 95%), menthol (CAS RN 89-78-1, purity ≥98.0%), and methyl eugenol (CAS RN 93-15-2, purity ≥98%) were purchased from Sig-ma-Aldrich (St. Louis, MO, USA). Benzyl alcohol (CAS RN 100-51-6, purity 99%), citral (CAS RN 5392-40-5, purity 95%), citronellol (CAS RN 106-22-9, purity 95%), geraniol (CAS RN 106-24-1, purity 97%), D-limonene (CAS RN 5989-27-5, purity 97%), and linalool (CAS RN 78-70-6, purity 97%) were purchased from Alfa Aesar (Ward Hill, MA, USA). Anisyl alcohol (CAS RN 105-13-5, purity >98.0%), benzyl benzoate (CAS RN 120-51-4, purity >99.0%), benzyl cinnamate (CAS RN 103-41-3, purity >98.0%), benzyl salicylate (CAS RN 118-58-1, purity >99.0%), coumarin (CAS RN 91-64-5, purity >99.0%), eugenol (CAS RN 97-53-0, purity >99.0%), isoeugenol (CAS RN 97-54-1, purity >97.0%), Lilial (CAS RN 80-54-6, purity >96.0%), and methyl 2-octynoate (CAS RN 111-12-6, purity >98.0%) were purchased from TCI (Tokyo, Japan). Primary standard stock solutions were prepared to be 1000 mg/L by diluting each standard substance with acetone. A secondary stock solution was prepared to have a concentration of 400 mg/L by mixing primary stock solutions, and a standard solution for the calibration curve was prepared by sequentially diluting the secondary stock solution. Standard stock solutions were stored in a deep freezer at −70 °C, and the standard solutions for calibration were newly prepared daily.

### 2.2. Sample Preparation

The most popular cigarette brands sold in the South Korean market were selected for this analysis. Twenty-four non-capsule cigarettes and 30 flavored capsule cigarettes were purchased at retail stores. Some capsule cigarettes had more than one type of capsule. An additive-free cigarette was used as a blank sample for preparing the matrix-match calibration curve and for calculating the method detection limit (MDL). However, blank samples for the analysis of capsules could not be used because there were no capsule cigarettes without flavoring ingredients in them and the capsules could not be divided. A detailed list of the cigarettes analyzed is available in the Supplementary Material (Table S1). Tobacco, filter tips, and capsules were separated from the whole cigarette. In total, 0.25 g of tobacco and 0.25 g of filter tips were used for extraction, and the entire capsule was extracted because it was impossible to divide the capsule. Thereafter, 25 mL of acetone was added to the tobacco and the filter tips, and 10 mL of acetone was added to the capsules. After that, samples of flavoring agents were extracted for 30 min at 220 rpm in an orbital shaker. The supernatant of this solution was filtered through a 0.45-µm PTFE syringe filter and then transferred into a 1.5 mL vial.

### 2.3. Method Validation

The limit of detection (LOD) and the limit of quantification (LOQ) were calculated as three- and tenfold the signal-to-noise ratio, respectively, and were considered MDL and LOQ for the analysis of capsules. The MDL and LOQ for the analysis of tobacco and filter tips were calculated as three and ten times the signal-to-noise ratio obtained for the spiked blank sample, respectively. The blank samples of tobacco and filter tips were spiked with standard solutions of three concentrations to contain the analyte at three concentrations, respectively. The solutions of the three concentrations (2.5 µg/g, 5.0 µg/g, and 50 µg/g) prepared in triplicate were analyzed together with the standard solution of the same concentration to calculate the recovery rate of the analyte, and the reproducibility was confirmed from the relative standard deviation between the results of the same sample type (tobacco or filter tips). Since capsules are immediately mixed with the solvent after bursting, the recovery rate for the capsule sample was not measured; however, the reproducibility of this method has been confirmed in previous studies [19].

### 2.4. GC-MS/MS Analysis

GC-MS/MS analysis was performed using Trace 1310 GC coupled with TSQ 8000 Evo (Thermo Fisher Scientific, Waltham, MA, USA). Separation was carried out on DB-HeavyWAX (30 m × 0.25 mm I.D., 0.25 μm film thickness) capillary column. One microliter of the sample was injected in splitless mode at 260 °C. The GC oven temperature was programmed from 40 °C (held 3 min) to 170 °C at 20 °C/min, to 180 °C at 2.5 °C/min (held 13 min), to 200 °C at 10 °C/min and 20 °C/min to 270 °C (held 10 min). Helium (99.999% purity) was used as the carrier gas (mobile phase) at a constant flow of 1 mL/min. Both the transfer line and ion source temperatures were maintained at 260 °C. Data were obtained in the selected reaction monitoring (SRM). The MS/MS transitions and the collision energies are indicated in the Supplementary Material (Table S2). The timed acquisition method was used to acquire timed SRM data, and the resulting total scan time, SRM time, and lowest dwell time were 0.3 s, 0.3 s, and 18.8 ms, respectively. Xcalibur Software 4.2 (Thermo Fisher Scientific, Waltham, MA, USA) was used for data acquisition and processing. Both the preparation of the calibration curve and the quantification of the sample were performed using Xcalibur Quan Browser. If the detected level (ng/mL) obtained by data processing was outside the range of the calibration curve, the sample was appropriately diluted and then re-analyzed. In addition, the detected level (ng/mL) was put into equation (1) to calculate the concentration (ng/g) of the flavoring agent:

Concentration (ng/g) = Detected Level (ng/mL) × Volume of Sample (mL)/Weight of Sample (g).(1)

Then, the concentration was entered into the equation (2) to calculate the content (µg/cigarette) of flavoring agent:

Content (µg/cigarette) = Concentration (ng/g) × Weight of Sample (g)/1000.(2)

### 2.5. Statistical Analysis

Statistical analysis was performed using SPSS 25 (IBM SPSS, IBM Corp., Armonk, NY, USA). The assumption of a normal distribution was tested using the Shapiro-Wilk test. The non-parametric Kruskal-Wallis test was performed to determine the difference in the levels of flavoring agents among the three groups of tobacco, filter tips, and capsules, and the Dunn–Bonferroni test was performed for the post hoc analysis. In addition, the Mann–Whitney U test was performed to compare the levels of flavoring agents of non-capsule and capsule cigarettes. The level of significance was set to α = 0.05. SIMCA 16.0.0.7738 (Sartorius, Germany) was used to perform principal component analysis (PCA).

### 2.6. Estimation of Inhalation Exposure from Allergenic Flavoring Agents among Cigarette Smokers

The levels (µg/cigarette) of flavoring agents detected in tobacco, filter tips, and capsules, which were separated from one cigarette, were summed to calculate the total content of flavoring agents per cigarette. The exposure level through inhalation was estimated from the average and maximum values of each substance. For substances detected below the LOQ, the value of the detected amount was replaced with the LOQ of each substance. However, substances that were not detected even in one product were not subject to exposure estimation. The following equation, adopted from Marano et al. (2018), was used to estimate the inhalation exposure [20,21]:

EC = C × N × ED × EF/(IR × AT).(3)

where: EC (mg/m^3^): exposure concentration via inhalation route

C (μg/cigarette): content of allergen per cigarette
N: average number of cigarettes smoked per day
ED (years): exposure duration
EF (days/years): exposure frequency
IR (m^3^/day): daily inhalation rate
AT (h): averaging time (ED × 365 days/year)

This equation has been previously used to estimate the concentration of inhalation exposure to cigarette smoke; however, in this study, inhalation exposure concentration was estimated by assuming that the total amount of flavoring agents in the cigarette are transferred to the main stream of smoke when smoking.

To estimate the exposure level among Korean cigarette smokers, parameters for exposure estimation were referenced from previous studies conducted on Koreans. The exposure duration (ED) was calculated as 69.5 by subtracting 13.2 [22], the age of onset of smoking, from the life expectancy of 82.7 in 2018 [23]. The averaging time was 25,367.5, which was obtained by multiplying the exposure duration (ED) by 365 (days/year). The daily inhalation rate (m^3^/day) was 14.25, which was the average of the long-term inhalation rates of men and women [24]. The average number of cigarettes smoked per day (N) was 13.2, which corresponds to the average daily smoking amount indicated in the Korea National Health and Nutrition Examination Survey [25].

### 2.7. Risk Characterization

Risk characterization ratios (RCRs) were determined by dividing the exposure amount by the DNEL for each substance. The inhalation DNEL value calculated for the general population was obtained from the International Uniform Chemical Information Database version 6 [18]. In general, the DNEL established for chronic exposure is the lowest DNEL; hence, for most substances and exposure scenarios, the long-term (chronic) DNEL is sufficient to control the risk [26]. In addition, even if the total exposure period for a day is clearly shorter than 24 h or exposure does not occur daily, a more conservative evaluation is possible by applying the chronic DNEL [27]. Because DNELs derived from local effects are larger or absent, the DNELs used in this study are derived from systemic effects.

## 3. Results

### 3.1. Method Validation

The method developed here was validated with respect to linearity, LOD, MDL, LOQ, accuracy, and precision. For each analyte, the LOD was 0.12–5.59 ng/mL and the LOQ was 0.41–18.62 ng/mL. The MDL and the LOQ for tobacco were 0.49–7.81 ng/mL and 1.63–26.03 ng/mL, respectively. The MDL and the LOQ for filter tips were 0.49–5.59 ng/mL and 1.63–18.63 ng/mL, respectively. In addition, the coefficient of determination (R^2^) of the calibration curve of all analytes was >0.999, thereby confirming the linearity of the analysis method (Supplementary Table S3).

**Figure 1:**
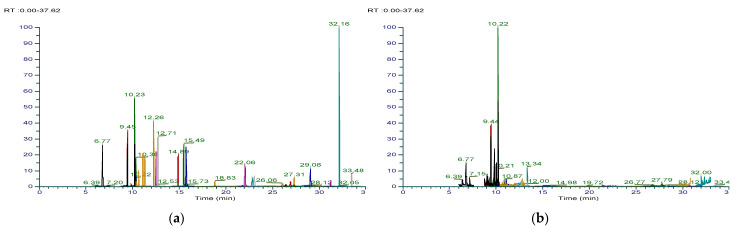

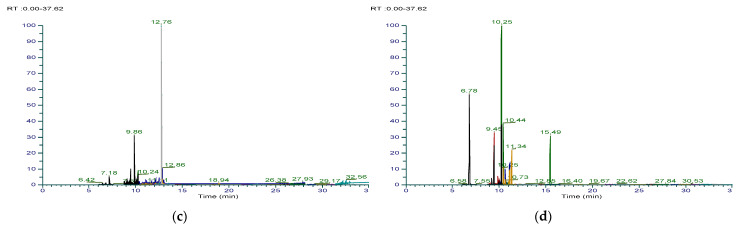
TIC of SRM of GC-MS/MS analysis: (**a**) standard mixture; (**b**) tobacco extraction solution; (**c**) filter tip extraction solution; (**d**) capsule extraction solution.

The accuracy and precision of the method were evaluated through the recovery test in unburned tobacco and filter tips. Recovery of all analytes was 81.0–108.3% for unburned tobacco and 80.3–104.4% for filter tips; therefore, it was possible to confirm the accuracy of the analysis method. In addition, since the relative standard deviation of the analysis results after repeated pre-treatment was <10% for all substances, the reproducibility of this analysis method was also confirmed (Supplementary Table S4). The total ion chromatograms (TICs) obtained as a result of the GC-MS/MS SRM analysis are shown in Figure 1.

### 3.2. Applicability of Analysis Method to Commercial Cigarettes

**Figure 2:**
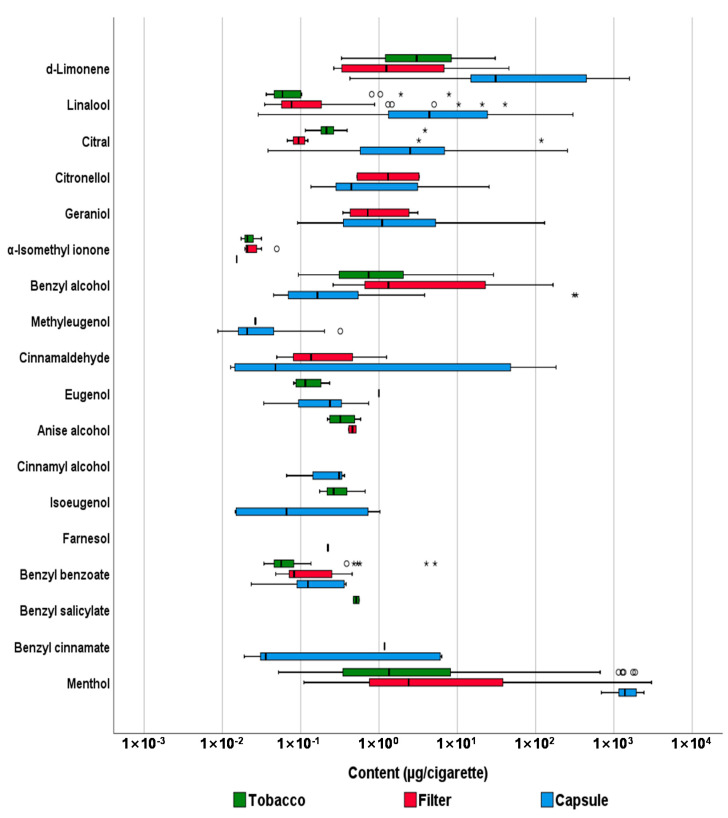
Comparison of content (µg/cigarette) of flavoring agents among unburned tobacco, filter tips, and capsules of cigarettes. The thick line inside the box represents the median, and the box edges represent the 25th percentile (Q1) and 75th percentile (Q3). The whiskers represent the minimum and maximum values (excluding outliers). The circles (○) represent the outliers (above Q3 + 1.5 × interquartile range), and the stars (*) represent the extreme values (above Q3 + 3 × interquartile range).

Tobacco, filter tips, and capsules separated from whole cigarettes were subjected to sample extraction with acetone, and the results of the GC-MS/MS analysis are summarized in Figure 2 and Table 1. The relevant concentration values (µg/cigarette) are shown in the Supplementary Material Table S5.

In the tobacco of the 54 cigarettes, 0.11–3.87 μg of citral was detected in all cigarettes, and 0.09–28.97 μg and 0.05–1868.14 μg of benzyl alcohol and menthol were detected in 52 and 53 cigarettes, respectively. In the 54 filter tips, 0.26–166.73 μg of benzyl alcohol was detected in all cigarettes, and 0.03–40.69 μg and 0.11–3011.88 μg of linalool and menthol were detected in 44 and 53 cigarettes, respectively. Among the 42 capsules, 689.47–2419.50 µg of menthol was detected in all cigarettes, and 0.43–1578.66 µg and 0.03–299.91 µg of D-limonene and linalool were detected in 40 and 41 cigarettes, respectively. Menthol was detected most frequently and was present at the highest levels in the three parts of the cigarettes, in the order of tobacco < filter tips < capsules, based on average values. In addition, the order of the detected amount for each part of the cigarettes was the same for D-limonene, benzyl alcohol, linalool, and citral. Substances detected at more than half the frequency in tobacco were citral, menthol, benzyl alcohol, and benzyl benzoate. In addition, benzyl alcohol, menthol, and linalool were detected in more than half of the filter tips, and menthol, linalool, D-limonene, citral, eugenol, geraniol, citronellol, and benzyl alcohol were detected in more than half of the capsules. In contrast, methyl 2-octynoate, hydroxycitronellal, Lilial, α-amylcinnamaldehyde, α-hexylcinnamaldehyde, coumarin, HICC, and α-amylcinnamyl alcohol were absent in the tobacco, filter tips, and capsules of all cigarettes assessed. Methyl eugenol, which was removed from the list of food additives owing to its carcinogenic effects on experimental animals [10], was detected in one filter tip and 12 capsules, and its maximum detected content was 0.32 µg. However, coumarin and HICC, which are restricted for use as flavoring agents in cosmetics and toys, were not detected in tobacco, filter tips, or capsules.

The Kruskal-Wallis test was performed to statistically compare the amount of flavoring agents in tobacco, filter tips, and capsules, and the Dunn-Bonferroni test was performed for the post hoc analysis (Table 2). We observed a significant difference in the levels of D-limonene, linalool, citral, benzyl alcohol, and menthol in the three parts of cigarettes (*p* < 0.001) because the amount of the analytes contained in the capsule was significantly higher than that in the two other parts of the cigarettes (*p* < 0.05). When comparing the levels of flavoring agents in the tobacco and filter tips, most of the analytes had a relatively but not significantly higher content of flavoring agents in the filter tips than in the tobacco. However, when the content of all the analytes was compared, it tended to be in the following order: tobacco < filter tips < capsules.

### 3.3. Comparison of Levels of Flavoring Agents between Non-Capsule and Capsule Cigarettes

A comparison of the levels of flavoring agents in capsule and non-capsule cigarettes was performed, and the results are summarized in Table 3. More substances were detected at a higher frequency in the tobacco and filter tips in capsule cigarettes, and their average detected levels were also higher in capsule cigarettes than in non-capsule cigarettes. However, the average content of benzyl alcohol, anise alcohol, and benzyl benzoate in tobacco was higher in non-capsule cigarettes, and benzyl salicylate was detected only in non-capsule cigarettes.

**Figure 3:**
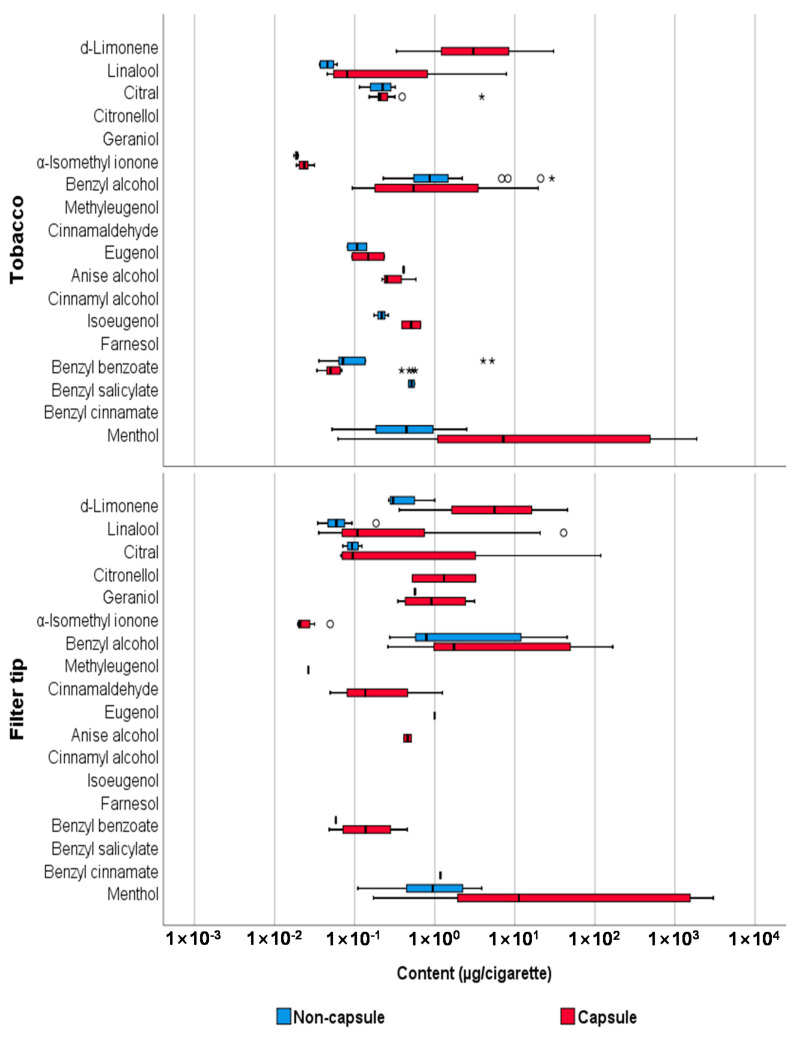
Comparison of content (µg/cigarette) of flavoring agents between capsule and non-capsule cigarettes. The thick line inside the box represents the median value, and the box edges represent the 25th percentile (Q1) and 75th percentile (Q3). The whiskers represent the minimum and maximum values (excluding outliers). The circles (○) represent the outliers (above Q3 + 1.5 × interquartile range), and the stars (*) represent the extreme values (above Q3 + 3 × interquartile range).

**Figure 4:**
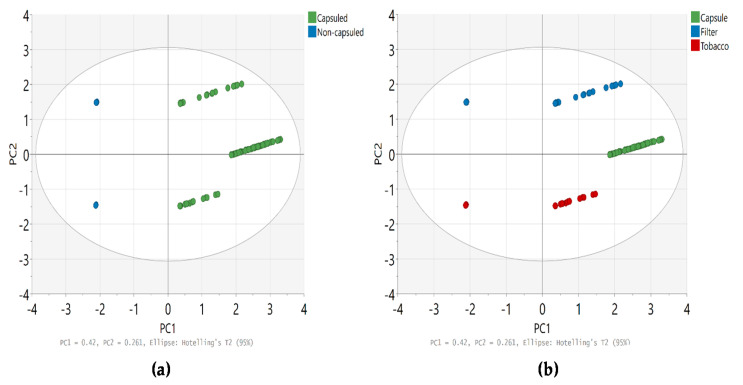
PCA for content (µg/cigarette) of flavoring agents in cigarettes (**a**) capsule cigarette vs. non-capsule cigarette; (**b**) tobacco, filter tip, and capsule.

The Mann-Whitney U test was performed to compare the content of flavoring agents between capsule and non-capsule cigarettes, and the results are summarized in Table 4. In summary, significant differences were observed in the levels of linalool, α-isomethyl ionone, benzyl benzoate, and menthol in tobacco between non-capsule and capsule cigarettes (*p* < 0.05); however, no significant difference was observed in the sum of the content of all substances (*p* = 0.583). Furthermore, the content of D-limonene, linalool, benzyl alcohol, and menthol in the filter tips of capsule cigarettes was significantly higher than that of non-capsule cigarettes (*p* < 0.05), and this difference was also confirmed when comparing the content of all analytes (*p* = 0.008). The distribution of the content of each analyte in tobacco, filter tips, and capsules is shown in Figure 3. In addition, PCA was performed, which revealed the distinct content of flavoring agents between capsule and non-capsule cigarettes and between tobacco, filter tips, and capsules (Figure 4). We found that the content of flavors can differentiate capsule cigarettes from non-capsule cigarettes as well as capsules from filter tips and tobacco.

### 3.4. Estimation of Inhalation Exposure and Risk Characterization

The average and maximum values of the inhalation exposure of the allergenic flavoring agents were calculated from the average and maximum values of the content of flavoring agents, which were estimated as 0.00004–1.450 mg/m^3^ and 0.00008–7.291 mg/m^3^, respectively (Table 5). Menthol, D-limonene, benzyl alcohol, linalool, and citral were estimated to have the highest inhalation exposure, as determined from the average and maximum levels of flavoring agents.

Estimated inhalation exposure was compared with the DNEL of each flavoring agent. Consequently, RCR was estimated to range from 2.6 × 10^−5^ to 1.8 × 10^−1^ in accordance with the average content, and menthol had the highest RCR. On considering the highest levels, the RCR ranged from 5.3 × 10^−5^ to 8.9 × 10^−1^, suggesting that the risk of all the analyzed flavoring agents is considered controlled. In particular, methyl eugenol was one of the substances with the lowest RCR upon risk characterization performed on the basis of the average and maximum values for inhalation exposure. However, some substances, such as menthol, linalool, and cinnamaldehyde, had an inhalation exposure exceeding one-tenth the DNEL (RCR > 0.1).

## 4. Discussion

In this study, we developed and validated an analysis method to simultaneously quantify 25 allergenic flavoring agents and menthol in cigarettes and used it on commercial cigarettes to quantify their levels in unburned tobacco, filter tips, and capsules. We found that this method was sufficiently accurate and reliable to analyze the flavoring allergens in cigarettes. The concentrations (in µg/g) of flavoring components in tobacco detected in this study were similar to those reported previously [13,14,15,16,17]. However, we could not compare our results with those of other studies with respect to the levels of flavoring agents in filter tips and capsules because no studies have analyzed allergenic flavorings in filter tips and capsules of cigarettes. Among flavoring agents restricted for use in food and cosmetics, methyl eugenol was detected in some cigarette filters and capsules; however, its content was relatively lower (the lowest content among the detected substances, as revealed from their median values) than that of other flavoring agents. The median concentration in the capsules (content per unit weight) was 1.0 mg/kg (=0.0001%), which was lower than the limit of the restricted substances in food and cosmetics (non-alcoholic beverages: 1 mg/kg, other leave-on products and oral products: 0.0002%) [5,9]. However, the highest amount of the detected substance was 15.0 mg/kg (=0.0015%), which exceeded the restricted limit.

Furthermore, we compared the flavoring agents in tobacco, filter tips, and capsules in cigarettes and observed a significant difference in the levels in the three parts. In addition, the results of the post hoc tests suggested that the content of flavoring agents in the capsules was significantly higher than that in the tobacco and filter tips. Furthermore, we compared the levels of flavoring agents in the tobacco and filter tips of non-capsule and capsule cigarettes and found that linalool and menthol were present at higher levels in the latter than in the former. In addition, it was confirmed that the sum of all analytes contained in the filter tips of capsule cigarettes was significantly higher than that of non-capsule cigarettes. This trend was also observed for menthol contained in tobacco and filter tips. However, the flavoring content of capsule cigarettes was not always higher than that of non-capsule cigarettes. For example, the content of benzyl benzoate was significantly higher in non-capsule cigarettes than in capsule cigarettes. The reason that the flavor component content of capsule cigarettes is higher than that of non-capsule cigarettes is presumed to be because the proportion of the capsules in the total flavor content in the cigarettes is very large. When statistically comparing the content of flavoring ingredients in tobacco, filter tips, and capsules (Table 2), it was confirmed that the content of flavoring ingredients in the capsules was always significantly higher than that in tobacco and filter tips. It was also confirmed in the result of the PCA (Figure 4). However, since non-capsule cigarettes do not contain capsules, the total flavor content of non-capsule cigarettes seems to be relatively lower than that of capsule cigarettes. Generally, consumers recognize the existence of flavoring agents in capsule cigarettes from their names or product information, but non-capsule cigarettes do not provide such information; hence, consumers easily mistake them for unflavored cigarettes. However, the present results suggest that non-capsule cigarettes also contain flavoring agents. In addition, some flavoring agents are contained at higher levels in non-capsule cigarettes than in capsule cigarettes, suggesting that even smokers of non-capsule cigarettes cannot completely rule out the possibility of exposure to allergenic flavorings.

Based on the analyzed content of flavoring agents, inhalation exposure was estimated on the basis of the content of flavoring agents in cigarettes, and the risk assessment was performed by comparing inhalation exposure with DNELs. Consequently, for all analytes, both mean and maximum exposure were below DNELs, suggesting that the risks posed by these substances were adequately controlled. However, on the risk assessment for all analytes, although exposure was estimated to be lower than DNELs, certain substances had the potential for exposure close to dangerous levels for some cigarettes. For example, the maximum inhalation exposures of menthol, linalool, and cinnamaldehyde exceeded one-tenth of the DNEL of each substance, and menthol exposure was especially high.

Of the approximately 7000 chemical agents found in cigarette smoke, WHO Tobacco Product Regulation has classified 39 priority toxicants [28], and the FDA has classified 93 chemicals as “harmful and potentially harmful constituents (HPHCs)” [29]. Furthermore, numerous studies have estimated smokers’ exposure to those toxicants, and the methodologies used for the exposure assessment can be largely divided into three categories: analysis of biomarkers in body fluids or exhaled air, machine smoking to simulate human smoking behavior, and estimation of exposure after analysis of nicotine and tar deposited on the filter tip after smoking [30]. However, these methodologies could not be used in this study because most analytes assessed here have not been assessed in other studies on cigarettes and factors (e.g., mouth-spill rate, absorption rate, and residual substances in the filter tips after smoking) required to estimate the exposure from smoking were unknown. Consequently, the risk assessment was conducted on the basis of the assumption that the entire amount of flavoring agents in the cigarettes is absorbed in the body. Hence, the risk of allergenic flavoring agents in cigarettes evaluated in this study may have been somewhat overestimated. However, this does not imply that the risk of exposure to flavoring allergens in cigarettes can be disregarded. The discordance between the DNEL and the level of exposure to allergens that may occur when smoking some cigarettes was not large enough to exclude the risk. Furthermore, the amount of flavoring agents contained in the filter tips and capsules was higher than that in tobacco, and the ingredients contained in these two parts are not thermally denatured or pyrolyzed upon combustion, unlike the ingredients in tobacco. Since there are few related studies, in this study, the risk caused by flavoring agents could only be evaluated under the assumption that the amount of flavoring agents absorbed in the body was the same as the amount contained in the cigarette. Therefore, further studies are required to conduct more accurate risk assessments, which will be pivotal to reduce the health risk from smoking.

## 5. Conclusions

Various flavored cigarettes are widely consumed; however, there are concerns regarding allergenic flavoring agents and subsequent adverse health effects. Hence, in this study, we developed and validated a GC-MS/MS–based method to simultaneously analyze 25 allergenic flavoring agents and menthol and applied this method to determine the content of flavoring agents in the tobacco, filter tips, and capsules in 54 commercial cigarettes. We found that not only tobacco but also filter tips and capsules contained various flavorings and that the content of the analyzed flavorings contained in the cigarettes was in the order of tobacco ≤ filter tips < capsules. Furthermore, even cigarettes without flavoring capsules contained flavoring agents, and their content was not always lower than that of capsule cigarettes. Since the individual exposure of all substances estimated using the obtained flavoring content did not exceed each DNEL, it is considered that flavoring agents are properly managed at present. However, since the maximum RCR calculated for menthol, linalool, and cinnamaldehyde was > 0.1, it is considered that continuous attention and proper management of the components are required for the safety of smokers.

## Figures and Tables

**Table 1:** Content (µg/cigarette) of flavoring agents in tobacco, filter tips, and capsules.

Compound	Tobacco (*n* = 54)						Filter Tip (*n* = 54)						Capsule (*n* = 42)					
	N	Mean	Median	SD	Min	Max	N	Mean	Median	SD	Min	Max	N	Mean	Median	SD	Min	Max
D-Limonene	7	8.50	3.02	10.93	0.33	30.49	11	8.85	1.24	14.68	0.26	45.62	40	265.07	30.87	379.35	0.43	1578.66
Linalool	21	0.60	0.06	1.68	0.04	7.83	44	1.92	0.08	6.85	0.03	40.69	41	33.31	4.39	63.04	0.03	299.91
Methyl 2-octynoate	0	ND	-	-	-	-	0	ND	-	-	-	-	0	ND	-	-	-	-
Citral	54	0.29	0.21	0.49	0.11	3.87	16	7.67	0.09	28.57	0.07	118.27	29	24.62	2.50	64.42	0.04	255.34
Citronellol	0	ND	-	-	-	-	2	1.89	1.89	1.36	0.53	3.25	25	2.81	0.44	5.45	0.14	25.49
Geraniol	0	ND	-	-	-	-	6	1.30	0.74	1.08	0.35	3.14	26	9.74	1.10	25.91	0.09	130.26
α-Isomethyl ionone	12	0.02	0.02	0.00	0.02	0.03	8	0.03	0.02	0.01	0.02	0.05	1	0.02	0.02	0.00	0.02	0.02
Benzyl alcohol	52	2.80	0.74	5.52	0.09	28.97	54	18.43	1.32	36.17	0.26	166.73	21	30.78	0.16	93.53	0.05	330.43
Hydroxycitronellal	0	ND	-	-	-	-	0	ND	-	-	-	-	0	ND	-	-	-	-
Methyl eugenol	0	ND	-	-	-	-	1	0.03	0.03	0.00	0.03	0.03	12	0.06	0.02	0.09	0.01	0.32
Cinnamaldehyde	0	ND	-	-	-	-	7	0.36	0.14	0.40	0.05	1.25	10	43.56	0.05	69.30	0.01	182.40
Lilial	0	ND	-	-	-	-	0	ND	-	-	-	-	0	ND	-	-	-	-
Eugenol	4	0.14	0.12	0.06	0.08	0.23	1	1.00	1.00	0.00	1.00	1.00	27	0.24	0.24	0.17	0.03	0.74
α-Amylcinnamaldehyde	0	ND	-	-	-	-	0	ND	-	-	-	-	0	ND	-	-	-	-
Anise alcohol	4	0.37	0.33	0.14	0.22	0.58	2	0.46	0.46	0.05	0.41	0.51	0	ND	-	-	-	-
Cinnamyl alcohol	0	ND	-	-	-	-	0	ND	-	-	-	-	3	0.25	0.31	0.13	0.07	0.36
Isoeugenol	5	0.34	0.26	0.18	0.17	0.66	0	ND	-	-	-	-	5	0.37	0.07	0.43	0.01	1.03
α-Hexylcinnamaldehyde	0	ND	-	-	-	-	0	ND	-	-	-	-	0	ND	-	-	-	-
Farnesol	0	ND	-	-	-	-	0	ND	-	-	-	-	2	0.22	0.22	0.00	0.22	0.22
Coumarin	0	ND	-	-	-	-	0	ND	-	-	-	-	0	ND	-	-	-	-
HICC	0	ND	-	-	-	-	0	ND	-	-	-	-	0	ND	-	-	-	-
α-Amylcinnamyl alcohol	0	ND	-	-	-	-	0	ND	-	-	-	-	0	ND	-	-	-	-
Benzyl benzoate	31	0.41	0.06	1.12	0.03	5.18	9	0.18	0.08	0.14	0.05	0.45	7	0.20	0.12	0.14	0.02	0.38
Benzyl salicylate	2	0.51	0.51	0.04	0.47	0.56	0	ND	-	-	-	-	0	ND	-	-	-	-
Benzyl cinnamate	0	ND	-	-	-	-	1	1.18	1.18	0.00	1.18	1.18	6	2.08	0.04	2.91	0.02	6.32
Menthol	53	191.07	1.34	448.81	0.05	1868.14	53	417.05	2.39	863.24	0.11	3011.88	42	1518.22	1378.95	450.78	689.47	2419.50

**Table 2:** Comparison of content (µg/cigarette) of flavoring agents detected in tobacco, filter tips, and capsules.

Compound	N	Kruskal-Wallis Test			Pairwise Comparisons Using Dunn-Bonferroni Test		
		H	df	*p*-Value		Mean Difference	*p*-Value
All analytes	757	56.721	2	<0.001 ***	tobacco-filter tip	−3.325	0.003 **
					tobacco-capsule	−7.511	<0.001 ***
					filter tip-capsule	−3.770	<0.001 ***
D-Limonene	58	17.768	2	<0.001 ***	tobacco-filter tip	−0.336	1.000
					tobacco-capsule	−2.669	0.023 *
					filter tip-capsule	−3.688	0.001 **
Linalool	106	50.156	2	<0.001 ***	tobacco-filter tip	−0.967	1.000
					tobacco-capsule	−5.861	<0.001 ***
					filter tip-capsule	−6.064	<0.001 ***
Citral	99	28.855	2	<0.001 ***	tobacco-filter tip	−3.308	0.006 **
					tobacco-capsule	−3.347	0.002 **
					filter tip-capsule	−5.293	<0.001 ***
Benzyl alcohol	127	25.216	2	<0.001 ***	tobacco-filter tip	−2.981	0.009 **
					tobacco-capsule	−2.582	0.029 *
					filter tip-capsule	−4.848	<0.001 ***
Menthol	148	62.913	2	<0.001 ***	tobacco-filter tip	−1.994	0.138
					tobacco-capsule	−7.713	<0.001 ***
					filter tip-capsule	−5.838	<0.001 ***

Asterisks (*, ** and ***) denote statistical significance with *p* < 0.05, *p* < 0.01 and *p* < 0.001, respectively.

**Table 3:** Comparison of content (µg/cigarette) of flavoring agents in non-capsule cigarettes and capsule cigarettes.

Compound	Non-Capsule Cigarette (*n* = 24)		Capsule Cigarette (*n* = 30)	
	Tobacco	Filter Tip	Tobacco	Filter Tip
D-Limonene	ND (0)	0.47 ± 0.31 (4)	8.50 ± 10.93 (7)	13.64 ± 16.60 (7)
Linalool	0.05 ± 0.01 (8)	0.07 ± 0.03 (17)	0.94 ± 2.06 (13)	3.08 ± 8.54 (37)
Methyl 2-octynoate	ND (0)	ND (0)	ND (0)	ND (0)
Citral	0.22 ± 0.06 (24)	0.10 ± 0.02 (10)	0.35 ± 0.66 (30)	20.30 ± 43.83 (6)
Citronellol	ND (0)	ND (0)	ND (0)	1.89 ± 1.36 (2)
Geraniol	ND (0)	0.57 (1)	ND (0)	1.45 ± 1.13 (5)
α-Isomethyl ionone	0.02 ± 0.00 (3)	ND (0)	0.02 ± 0.00 (9)	0.03 ± 0.01 (8)
Benzyl alcohol	3.63 ± 7.14 (22)	8.40 ± 13.27 (24)	2.19 ± 3.81 (30)	26.46 ± 45.49 (30)
Hydroxycitronellal	ND (0)	ND (0)	ND (0)	ND (0)
Methyl eugenol	ND (0)	ND (0)	ND (0)	0.03 (1)
Cinnamaldehyde	ND (0)	ND (0)	ND (0)	0.36 ± 0.40 (7)
Lilial	ND (0)	ND (0)	ND (0)	ND (0)
Eugenol	0.11 ± 0.03 (2)	ND (0)	0.16 ± 0.07 (2)	1.00 (1)
α-Amylcinnamaldehyde	ND (0)	ND (0)	ND (0)	ND (0)
Anise alcohol	0.41 (1)	ND (0)	0.35 ± 0.16 (3)	0.46 ± 0.05 (2)
Cinnamyl alcohol	ND (0)	ND (0)	ND (0)	ND (0)
Isoeugenol	0.22 ± 0.04 (3)	ND (0)	0.53 ± 0.14 (2)	ND (0)
α-Hexylcinnamaldehyde	ND (0)	ND (0)	ND (0)	ND (0)
Farnesol	ND (0)	ND (0)	ND (0)	ND (0)
Coumarin	ND (0)	ND (0)	ND (0)	ND (0)
HICC	ND (0)	ND (0)	ND (0)	ND (0)
α-Amylcinnamyl alcohol	ND (0)	ND (0)	ND (0)	ND (0)
Benzyl benzoate	1.08 ± 1.90 (9)	0.06 (1)	0.13 ± 0.17 (22)	0.19 ± 0.14 (8)
Benzyl salicylate	0.51 ± 0.04 (2)	ND (0)	ND (0)	ND (0)
Benzyl cinnamate	ND (0)	ND (0)	ND (0)	1.18 (1)
Menthol	0.75 ± 0.77 (23)	1.35 ± 1.04 (23)	336.98 ± 553.89 (30)	735.76 ± 1040.40 (30)

**Table 4:** Comparison of flavoring agents detected in capsule cigarettes and non-capsule cigarettes.

Compound	Category	Tobacco (*n* = 54)					Filter (*n* = 54)				
		N	Mean	Sum	Mann-Whitney U	*p*-Value	N	Mean	Sum	Mann-Whitney U	*p*-Value
All analytes	Non-capsule	97	119.93	11,633	6880	0.583	80	93.31	7465	4225	0.008 **
	Capsule	148	125.01	18,502			135	116.70	15,755		
D-Limonene	Non-capsule	0	0.00	0	-	-	4	2.75	11	1	0.014 *
	Capsule	7	4.00	28			7	7.86	55		
Linalool	Non-capsule	8	6.38	51	15	0.007 **	17	14.76	251	98	0.002 **
	Capsule	13	13.85	180			27	27.37	739		
Citral	Non-capsule	24	25.88	621	321	0.497	10	8.30	83	28	0.828
	Capsule	30	28.80	864			6	8.83	53		
α-Isomethyl ionone	Non-capsule	3	2.67	8	2	0.033 *	0	0.00	0	-	-
	Capsule	9	7.78	70			8	4.50	36		
Benzyl alcohol	Non-capsule	22	30.00	660	253	0.154	24	22.46	539	239	0.035 *
	Capsule	30	23.93	718			30	31.53	946		
Benzyl benzoate	Non-capsule	9	21.22	191	52	0.041 *	1	2.00	2	1	0.245
	Capsule	22	13.86	305			8	5.38	43		
Menthol	Non-capsule	23	18.26	420	144	<0.001 ***	23	17.30	398	122	<0.001 ***
	Capsule	30	33.70	1011			30	34.43	1033		

Asterisks (*, ** and ***) denote statistical significance with *p* < 0.05, *p* < 0.01 and *p* < 0.001, respectively.

**Table 5:** Risk assessment of allergenic flavoring agents in flavored cigarettes.

Compound	DNEL ^a^ (mg/m^3^)	Average			Maximum		
		Content (μg/Cigarette)	Exposure (mg/m^3^)	RCR^b^	Content (μg/Cigarette)	Exposure (mg/m^3^)	RCR
D-Limonene	16.6	119.47	0.11067	6.7 × 10^−3^	818.21	0.75792	4.6 × 10^−2^
Linalool	0.7	23.22	0.02151	3.1 × 10^−2^	301.86	0.27962	4.0 × 10^−1^
Citral	2.7	15.12	0.01400	5.2 × 10^−3^	255.67	0.23683	8.8 × 10^−2^
Citronellol	47.8	1.60	0.00149	3.1 × 10^−5^	26.15	0.02422	5.1 × 10^−4^
Geraniol	47.8	4.27	0.00396	8.3 × 10^−5^	130.58	0.12096	2.5 × 10^−3^
α-Isomethyl ionone	1.45	0.04	0.00004	2.6 × 10^−5^	0.08	0.00008	5.3 × 10^−5^
Benzyl alcohol	5.4	33.10	0.03066	5.7 × 10^−3^	474.41	0.43945	8.1 × 10^−2^
Methyl eugenol	1.74	0.05	0.00005	2.6 × 10^−5^	0.35	0.00033	1.9 × 10^−4^
Cinnamaldehyde	1.09	8.15	0.00755	6.9 × 10^−3^	182.95	0.16947	1.6 × 10^−1^
Eugenol	5.22	0.27	0.00025	4.8 × 10^−5^	1.38	0.00128	2.5 × 10^−4^
Anise alcohol	0.37	0.33	0.00031	8.3 × 10^−4^	1.16	0.00107	2.9 × 10^−3^
Cinnamyl alcohol	0.465	0.09	0.00008	1.8 × 10^−4^	0.43	0.00040	8.5 × 10^−4^
Isoeugenol	5.22	0.33	0.00031	5.9 × 10^−5^	1.27	0.00118	2.3 × 10^−4^
Farnesol	0.457	0.86	0.00080	1.7 × 10^−3^	1.12	0.00103	2.3 × 10^−3^
Benzyl benzoate	1.25	0.33	0.00031	2.5 × 10^−4^	5.24	0.00485	3.9 × 10^−3^
Benzyl salicylate	1.37	0.61	0.00057	4.1 × 10^−4^	0.82	0.00076	5.5 × 10^−4^
Benzyl cinnamate	1.74	0.33	0.00030	1.7 × 10^−4^	6.41	0.00593	3.4 × 10^−3^
Menthol	8.17	1565.01	1.44969	1.8 × 10^−1^	7870.83	7.29087	8.9 × 10^−1^

^a^ DNEL: Derived no-effect level. ^b^ RCR: Risk characterization ratio.

## Data Availability

Data is contained within the article or Supplementary Material.

## Supplementary Materials

The following are available online at https://www.mdpi.com/article/10.3390/toxics9040087/s1, Table S1: Non-capsule cigarettes and capsule cigarettes selected for analysis, Table S2: Optimized GC-MS/MS conditions for analyzing flavoring agents in cigarettes, Table S3: Method validation (MDL, LOQ, and linearity) for analyzing allergenic flavoring agents, Table S4: Recovery and reproducibility of analysis of allergenic flavoring agents in cigarette matrix, Table S5. Concentration (µg/g) of flavoring agents in tobacco, filter tips, and capsules.
