# Supplementary material for: Development of a Method for Simultaneous Analysis of Allergenic Flavoring Agents in Cigarettes and Quantitative Risk Assessment for Consumer Safety
Source: Toxics. 2021 Apr 18;9(4):87. doi: 10.3390/toxics9040087 (PMC8072964; doi:10.3390/toxics9040087)
Supplement: Supplementary file 1 [file toxics-09-00087-s001.zip › toxics-1160558-supplementary.pdf]

Supplementary Materials: Development of A Method for Simultaneous Analysis of Allergenic Flavoring Agents in Cigarettes and Quantitative Risk assessment for Consumer Safety

Dae Yong Jang, Hyung Soo Kim, Eun Chul Pack, Ye Ji Koo, Kyung Min Lim and Dal Woong Choi

Table S1. Non-capsule cigarettes and capsule cigarettes selected for analysis.

No.	Brand	Category	Cigarette Size	Number of Capsules	Sub-Brand	Flavor Type
1	A	Non-capsule	King	None	Plus	-
2			King	None	Origin	-
3		Capsule	King	1 EA	Ice Kula	Mint
4			King	1 EA	Rula	Banana
5			King	1 EA (Random, 1 out of 5)	Mola	Fruit
6			King	1 EA	Ice Jack	Mint
7	B	Non-capsule	Slim	None	Prime	-
8			Slim	None	Soo 0.1	-
9			Slim	None	Soo	-
10			Slim	None	Sense 1	-
11			Slim	None	One	-
12			Slim	None	Classic	-
13		Capsule	Slim	1 EA	Change 1mg	Mint
14			Slim	1 EA	Change W	Wine
15			Slim	1 EA	Change Up	Orange
16			Slim	1 EA	Change LiNN	Mint
17			Slim	1 EA	Change Bing	Sweet
18			Slim	1 EA	Change Frozen	Mint
19			Slim	1 EA	Change Himalaya	Mint
20			Slim	1 EA (Random, 1 out of 5)	Change Secret	Fruit
21			Slim	1 EA	Change 4mg	Mint
22	C	Non-capsule	King	None	Gold	-
23			King	None	Silver	-
24			King	None	Medium	-
25			King	None	Red	-
26		Capsule	King	1 EA	Hybrid 1	Mint
27			King	1 EA	Ice Blast One	Mint

28			King	1 EA	Hybrid 5	Mint
29			King	2 EA	Zing Fusion Double	Orange
30			King	1 EA	Ice Blast	Mint
31	D	Non-capsule	King	None	6mg	-
32			Slim	None	Fine Cut Master 1mg	-
33			Slim	None	Fine Cut Master 3mg	-
34			King	None	1mg	-
35			Slim	None	Fine Cut 1mg	-
36			King	None	3mg	-
37		Capsule	King	1 EA	Switch one	Mint
38			Slim	1 EA	Fine Cut Switch	Mint
39			Slim	2 EA	Fine Cut Mellow Crush	Mellon, Mint
40			Slim	2 EA	Fine Cut Tropical Crush	Pineapple, Mint
41			King	2 EA	Summer Crush	Watermelon, Orange
42			King	1 EA	Switch 6mg	Mint
43	E	Non-capsule	King	None	Sky Blue	-
44			King	None	LSS One	-
45			King	None	One	-
46			King	None	Wind Blue	-
47			King	None	LSS Wind Blue	-
48			King	None	Original	-
49		Capsule	King	1 EA	LBS Yellow 1mg	Lemon
50			King	1 EA	LBS Blue	Mint
51			King	1 EA	LBS Mix Green	Fruit
52			King	1 EA	LBS Yellow 3mg	Lemon
53			King	1 EA	LBS Tropical Mix	Melon, Mint
54			King	1 EA	LBS Purple	Grape
Blank	C	Non-capsule	Slim	None	Zero Additive	-
	Total number of tobacco or filter tips: 55 EA				Total number of capsules: 42 EA	

Table S2. Optimized GC-MS/MS conditions for analyzing flavoring agents in cigarettes.

Compound	Retention Time (min)	SRM Transition 1			SRM Transition 2		
		Precursor Ion(m/z)	Product Ion (m/z)	Collision Energy	Precursor Ion (m/z)	Product Ion (m/z)	Collision Energy
D-Limonene	6.39	68.1	53.1	10	93.1	77.0	12
Linalool	9.45	93.1	77.1	12	121.1	93.1	6
Methyl 2-octynoate	10.36	95.1	67.1	8	123.1	81.1	8
Citral	10.65; 11.11	94.1	79.1	8	69.1	41.1	6
Citronellol	11.32	95.1	67.1	8	69.1	41.1	6
Geraniol	12.26	69.1	41.1	6	93.1	77.0	12
α-Isomethyl ionone	12.53	150.1	107.1	6	135.2	91.1	14
Benzyl alcohol	12.71	108.1	79.1	12	79.1	77.1	10
Hydroxycitronellal	13.62	71.1	43.1	6	59.0	31.1	10
Methyl eugenol	14.89	178.1	107.1	14	163.1	107.1	6
Cinnamaldehyde	15.49	131.0	77.0	22	103.1	77.1	10
Lilial	15.72	189.1	131.1	10	204.1	189.2	8
Eugenol	18.83	164.1	131.1	10	131.1	103.1	10
α-Amylcinnamaldehyde	22.06	129.1	128.1	18	202.1	129.1	6
Anise alcohol	22.84	138.0	109.1	6	137.0	109.0	6
Cinnamyl alcohol	23.04	134.1	92.1	6	115.1	89.1	14
Isoeugenol	22.04; 26.45	164.1	131.0	10	149.1	121.1	6
α-Hexylcinnamaldehyde	26.93	216.2	129.1	6	117.1	115.1	8
Farnesol	26.07; 27.31	81.1	79.2	10	93.1	77.1	10
Coumarin	29.08	118.0	90.1	10	146.0	118.1	10
HICC	29.99; 30.23	105.1	79.0	10	136.1	79.1	8
α-Amylcinnamyl alcohol	31.25	133.1	55.0	8	91.1	65.0	14
Benzyl benzoate	32.16	105.0	77.1	12	91.1	65.1	12
Benzyl salicylate	33.48	228.1	91.1	6	91.1	65.1	14
Benzyl cinnamate	36.76	131.0	103.1	8	91.1	65.1	12
Menthol	10.25	95.1	67.1	8	123.1	81.1	8

Table S3. Method validation (MDL, LOQ, and linearity) for analyzing allergenic flavoring agents.

Compound	LOD (ng/ml)	MDL (ng/ml)		LOQ (ng/ml)			Calibration Range (ng/ml)	Linearity (R^2)
		Tobacco	Filter tip	Capsule	Tobacco	Filter tip		
D-Limonene	3.91	7.81	3.91	13.03	26.03	13.03	13.02 - 2,000	0.9999
Linalool	0.49	0.98	1.95	1.63	3.27	6.50	1.63 - 125;<br>125 - 2,000	0.9999;<br>0.9999
Methyl 2-octynoate	0.98	3.91	3.91	3.27	13.03	13.03	3.26 - 125	0.9998
Citral	0.98	1.95	0.98	3.27	6.50	3.27	3.26 - 1,000	0.9999
Citronellol	3.91	3.91	3.91	13.03	13.03	13.03	13.02 - 2,000	0.9999
Geraniol	1.95	3.91	3.91	6.50	13.03	13.03	6.51 - 62.5;<br>62.5 - 2,000	0.9998;<br>0.9997
α-Isomethyl ionone	0.24	0.49	3.91	0.80	1.63	13.03	0.81 - 125	0.9999
Benzyl alcohol	0.98	1.95	3.91	3.27	6.50	13.03	3.26 - 2,000	0.9999
Hydroxycitronellal	3.91	3.91	3.91	13.03	13.03	13.03	13.02 - 250	0.9996
Methyl eugenol	0.24	1.95	0.98	0.80	6.50	3.27	0.81 - 125	0.9999
Cinnamaldehyde	0.24	0.49	0.49	0.80	1.63	1.63	0.81 - 125	0.9999
Lilial	0.12	0.49	0.98	0.40	1.63	3.27	0.41 - 62.5	0.9998
Eugenol	0.98	0.98	3.91	3.27	3.27	13.03	3.26 - 250	0.9999
α-Amylcinnamaldehyde	0.98	0.98	1.95	3.27	3.27	6.50	3.26 - 125	0.9999
Anise alcohol	1.95	1.95	1.95	6.50	6.50	6.50	6.51 - 500	0.9999
Cinnamyl alcohol	0.49	0.98	0.49	1.63	3.27	1.63	1.63 - 250	0.9998
Isoeugenol	1.82	1.82	1.82	6.07	6.07	6.07	6.08 - 116.75	0.9999
α-Hexylcinnamaldehyde	0.98	0.98	0.98	3.27	3.27	3.27	3.26 - 125	0.9997
Farnesol	5.59	5.59	5.59	18.63	18.63	18.63	18.62 - 179	0.9999
Coumarin	0.98	1.95	3.91	3.27	6.50	13.03	3.26 - 125	0.9997
HICC	0.24	0.98	1.95	0.80	3.27	6.50	0.81 - 62.5	0.9998
α-Amylcinnamyl alcohol	1.95	1.95	3.91	6.50	6.50	13.03	6.51 - 125	0.9999
Benzyl benzoate	0.49	0.49	0.49	1.63	1.63	1.63	1.63 - 500	0.9998
Benzyl salicylate	3.91	3.91	3.91	13.03	13.03	13.03	13.02 - 250	0.9999
Benzyl cinnamate	0.49	2.44	0.98	1.63	8.13	3.27	1.63 - 125	0.9999
Menthol	0.49	0.98	1.95	1.63	3.27	6.50	1.63 - 156.25;<br>156.25 - 5,000	0.9999;<br>0.9999

MDL, Method detection limit; LOQ, Limit of quantitation.

Table S4. Recovery and reproducibility of analysis of allergenic flavoring agents in cigarette matrix.

Compound	Tobacco (*n* = 3, for individual concentrations)						Filter Tip (n = 3, for individual concentrations)					
	Recovery (%)			Reproducibility (% RSD)			Recovery (%)			Reproducibility (% RSD)		
	2.5 μg/g	5.0 μg/g	50 μg/g	2.5 μg/g	5.0 μg/g	50 μg/g	2.5 μg/g	5.0μg/g	50 μg/g	2.5 μg/g	5.0 μg/g	50 μg/g
D-Limonene	94.2 (2.2%)	91.0 (4.8%)	93.0 (1.0%)	1.81	3.95	0.81	92.9 (2.5%)	97.3 (2.3%)	93.1 (1.3%)	2.02	1.89	1.03
Benzyl alcohol	89.7 (4.8%)	101.4 (4.5%)	92.9 (1.8%)	3.88	3.68	1.49	94.3 (1.9%)	98.4 (2.2%)	93.2 (2.4%)	1.52	1.82	1.96
Linalool	95.5 (4.2%)	104.0 (0.8%)	102.7 (0.6%)	3.45	0.68	0.46	92.9 (1.2%)	99.2 (2.8%)	96.8 (1.5%)	0.95	2.31	1.22
Methyl 2-octynoate	89.0 (2.5%)	96.4 (1.1%)	106.4 (0.4%)	2.01	0.89	0.34	86.0 (6.8%)	90.6 (5.3%)	100.5 (1.5%)	5.56	4.35	1.24
Citronellol	103.2 (4.6%)	103.2 (5.4%)	108.3 (0.6%)	3.73	4.43	0.48	90.5 (0.7%)	99.6 (3.7%)	101.1 (1.6%)	0.60	3.05	1.32
Citral	89.5 (5.3%)	95.9 (4.3%)	105.1 (2.0%)	4.28	3.49	1.67	94.4 (0.8%)	104.4 (2.1%)	100.3 (1.7%)	0.65	1.72	1.40
Geraniol	88.3 (1.8%)	98.1 (2.9%)	99.5 (3.1%)	1.45	2.39	2.50	91.5 (2.1%)	97.2 (5.1%)	91.1 (3.0%)	1.72	4.19	2.45
Cinnamaldehyde	101.4 (8.0%)	102.6 (3.3%)	97.9 (0.6%)	6.56	2.71	0.45	100.1 (0.9%)	102.8 (2.7%)	96.6 (1.4%)	0.70	2.24	1.13
Anise alcohol	91.5 (3.1%)	93.8 (4.9%)	90.8 (5.0%)	2.51	3.96	4.11	87.0 (1.4%)	91.2 (4.1%)	97.0 (2.8%)	1.18	3.38	2.28
Hydroxycitronellal	101.3 (1.8%)	103.5 (3.0%)	105.2 (0.9%)	1.49	2.41	0.71	95.1 (1.5%)	103.3 (3.4%)	103.8 (0.9%)	1.20	2.76	0.70
Cinnamyl alcohol	89.3 (9.7%)	96.4 (5.2%)	99.3 (1.9%)	7.95	4.20	1.55	94.9 (1.0%)	92.3 (2.6%)	96.9 (1.4%)	0.77	2.13	1.12
Eugenol	102.9 (1.6%)	105.8 (1.1%)	101.0 (0.4%)	1.33	0.86	0.36	99.4 (2.0%)	102.8 (2.2%)	101.9 (3.9%)	1.66	1.76	3.14
Methyl eugenol	103.7 (2.7%)	104.8 (3.2%)	101.3 (0.2%)	2.18	2.61	0.14	97.9 (4.8%)	95.9 (1.6%)	97.1 (1.3%)	3.93	1.33	1.07
Coumarin	102.5 (3.3%)	98.7 (5.1%)	101.4 (0.2%)	2.72	4.16	0.12	100.7 (2.4%)	96.9 (0.8%)	98.9 (0.9%)	1.95	0.65	0.76
Isoeugenol	99.9 (4.5%)	100.9 (3.1%)	102.8 (2.0%)	3.68	2.53	1.62	95.9 (3.5%)	94.7 (1.4%)	91.2 (5.5%)	2.84	1.12	4.50
α-Isomethyl ionone	106.6 (2.4%)	101.9 (1.8%)	106.0 (0.3%)	0.39	1.40	0.69	101.1 (2.7%)	100.7 (2.1%)	101.1 (1.6%)	0.39	1.40	0.69

Lilial	93.4 (2.2%)	96.5 (4.1%)	101.2 (0.9%)	1.80	3.35	0.73	102.9 (1.0%)	95.1 (3.4%)	97.8 (1.4%)	0.83	2.81	1.12
α-Amylcinnamaldehyde	87.9 (8.4%)	100.6 (2.0%)	98.5 (2.2%)	6.82	1.59	1.79	85.9 (2.8%)	97.2 (1.4%)	96.8 (2.1%)	2.27	1.17	1.68
HICC	95.7 (5.3%)	101.3 (1.4%)	104.9 (3.3%)	4.30	1.13	2.67	97.3 (1.7%)	102.5 (5.6%)	103.9 (1.3%)	1.40	4.60	1.09
α-Amylcinnamyl alcohol	95.4 (4.3%)	104.2 (3.3%)	104.3 (1.5%)	3.53	2.71	1.25	92.8 (2.4%)	98.4 (4.6%)	103.1 (1.8%)	1.97	3.71	1.49
Farnesol	95.2 (5.6%)	91.7 (3.2%)	100.4 (2.2%)	4.55	2.60	1.75	94.1 (2.6%)	80.3 (4.6%)	87.7 (4.9%)	2.12	3.76	3.98
α-Hexylcinnamaldehyde	97.7 (2.1%)	100.6 (4.2%)	105.4 (1.8%)	1.71	3.41	1.45	95.1 (1.9%)	98.3 (4.1%)	100.8 (1.1%)	1.55	3.32	0.92
Benzyl benzoate	82.8 (5.7%)	90.9 (0.7%)	86.0 (3.0%)	4.66	0.58	2.48	92.1 (2.0%)	93.4 (1.1%)	83.8 (1.3%)	1.65	0.87	1.09
Benzyl salicylate	81.0 (3.0%)	82.8 (3.4%)	104.7 (1.7%)	2.44	2.74	1.37	90.2 (2.9%)	80.5 (3.4%)	97.0 (1.3%)	2.39	2.74	1.08
Benzyl cinnamate	85.6 (8.4%)	94.2 (4.8%)	102.6 (3.0%)	6.87	3.91	2.48	87.3 (8.7%)	90.9 (5.8%)	96.1 (1.8%)	7.09	4.70	1.43

Table S5. Concentration (μg/g) of flavoring agents in tobacco, filter tips, and capsules**.**

Compound	Tobacco (*n* = 54)						Filter tip (*n* = 54)						Capsule (*n* = 42)					
	N	Mean	Median	SD	Min	Max	N	Mean	Median	SD	Min	Max	N	Mean	Median	SD	Min	Max
D-Limonene	7	21.0	9.4	23.9	1.0	63.2	11	86.9	14.9	136.8	1.3	375.1	40	18,561.9	1,868.8	25,578.1	20.3	81,795.7
Linalool	21	1.9	0.1	5.5	0.1	25.1	44	21.7	0.5	81.3	0.2	472.0	41	2,152.3	213.3	3,893.0	2.0	14,558.8
Methyl 2-octynoate	0	-	-	-	-	-	0	-	-	-	-	-	0	-	-	-	-	-
Citral	54	0.7	0.5	1.6	0.2	12.4	16	88.0	0.7	331.6	0.4	1,372.1	29	1,254.2	203.3	2,994.2	3.1	11,931.8
Citronellol	0	-	-	-	-	-	2	19.6	19.6	13.5	6.1	33.0	25	180.6	22.7	289.4	12.2	1,237.6
Geraniol	0	-	-	-	-	-	6	13.7	7.3	12.6	2.8	36.4	26	548.8	103.3	1,297.8	7.7	6,323.2
α-Isomethyl ionone	12	0.04	0.04	0.01	0.03	0.07	8	0.2	0.2	0.1	0.1	0.4	1	1.3	1.3	-	1.3	1.3
Benzyl alcohol	52	5.5	1.3	10.4	0.2	55.6	54	138.4	14.4	304.3	1.7	1,761.9	21	2,151.7	11.1	6,544.7	1.8	23,269.9
Hydroxycitronellal	0	-	-	-	-	-	0	-	-	-	-	-	0	-	-	-	-	-
Methyleugenol	0	-	-	-	-	-	1	0.3	0.3	-	0.3	0.3	12	2.9	1.0	4.3	0.4	15.0
Cinnamaldehyde	0	-	-	-	-	-	7	2.5	1.1	2.5	0.5	8.2	10	2,035.1	4.3	3,231.1	0.6	8,523.5
Lilial	0	-	-	-	-	-	0	-	-	-	-	-	0	-	-	-	-	-
Eugenol	4	0.2	0.2	0.1	0.1	0.4	1	8.9	8.9	-	8.9	8.9	27	13.6	14.4	7.7	1.6	34.8
α-Amylcinnamaldehyde	0	-	-	-	-	-	0	-	-	-	-	-	0	-	-	-	-	-
Anise alcohol	4	0.8	0.5	0.6	0.4	1.8	2	4.8	4.8	1.1	3.7	6.0	0	-	-	-	-	-
Cinnamyl alcohol	0	-	-	-	-	-	0	-	-	-	-	-	3	11.5	14.4	6.0	3.1	16.9
Isoeugenol	5	0.6	0.4	0.3	0.3	1.1	0	-	-	-	-	-	5	17.3	3.1	19.9	0.8	48.2
α-Hexylcinnamaldehyde	0	-	-	-	-	-	0	-	-	-	-	-	0	-	-	-	-	-
Farnesol	0	-	-	-	-	-	0	-	-	-	-	-	2	10.6	10.6	0.2	10.4	10.8
Coumarin	0	-	-	-	-	-	0	-	-	-	-	-	0	-	-	-	-	-
HICC	0	-	-	-	-	-	0	-	-	-	-	-	0	-	-	-	-	-
α-Amylcinnamyl alcohol	0	-	-	-	-	-	0	-	-	-	-	-	0	-	-	-	-	-
Benzyl benzoate	31	0.7	0.1	1.8	0.1	8.1	9	1.3	0.6	1.1	0.3	3.7	7	14.8	6.8	12.7	1.6	36.5
Benzyl salicylate	2	0.9	0.9	0.1	0.7	1.0	0	-	-	-	-	-	0	-	-	-	-	-
Benzyl cinnamate	0	-	-	-	-	-	1	8.6	8.6	-	8.6	8.6	6	145.7	2.9	203.1	0.9	439.2
Menthol	53	463.8	2.4	1,211.4	0.1	5,949.5	53	3,633.5	17.6	7,678.9	1.2	31,006.5	42	98,948.6	96,829.4	31,755.3	42,307.9	168,826.6

n, The number of total samples; N, The number of sample containing flavoring agent; SD, Standard deviation.
